# Oxidative stress–induced mitochondrial dysfunction drives inflammation and airway smooth muscle remodeling in patients with chronic obstructive pulmonary disease

**DOI:** 10.1016/j.jaci.2015.01.046

**Published:** 2015-09

**Authors:** Coen H. Wiegman, Charalambos Michaeloudes, Gulammehdi Haji, Priyanka Narang, Colin J. Clarke, Kirsty E. Russell, Wuping Bao, Stelios Pavlidis, Peter J. Barnes, Justin Kanerva, Anton Bittner, Navin Rao, Michael P. Murphy, Paul A. Kirkham, Kian Fan Chung, Ian M. Adcock, COPDMAP, Christopher E. Brightling, Donna E. Davies, Donna K. Finch, Andrew J. Fisher, Alasdair Gaw, Alan J. Knox, Ruth J. Mayer, Michael Polkey, Michael Salmon, David Singh

**Affiliations:** a Airway Disease Section, National Heart & Lung Institute, Imperial College London, London, United Kingdom; b Janssen Research & Development, High Wycombe, United Kingdom; c Janssen Research & Development LLC, San Diego, Calif; d MRC Mitochondrial Biology Unit, Cambridge, United Kingdom

**Keywords:** Ozone, inflammation, airway smooth muscle, mitochondria, chronic obstructive pulmonary disease, airway hyperresponsiveness, oxidative stress, antioxidant, proliferation, MitoQ, AHR, Airway hyperresponsiveness, ASM, Airway smooth muscle, ATP, Adenosine triphosphate, BAL, Bronchoalveolar lavage, COPD, Chronic obstructive pulmonary disease, dTPP, Decyltriphenylphosphonium bromide, GOLD, Global Initiative for Chronic Obstructive Lung Disease, JC-1, 5,5′,6,6′-Tetrachloro-1,1′,3,3′-tetraethylbenzimidazolylcarbocyanine iodide, KC, Keratinocyte-derived cytokine, −logPC_100_, Concentration of acetylcholine that increased lung resistance by 100%, ΔΨm, Mitochondrial membrane potential, NAC, N-acetylcysteine, NO, Nitric oxide, OCR, Oxygen consumption rate, R_L_, Lung resistance, ROS, Reactive oxygen species

## Abstract

**Background:**

Inflammation and oxidative stress play critical roles in patients with chronic obstructive pulmonary disease (COPD). Mitochondrial oxidative stress might be involved in driving the oxidative stress–induced pathology.

**Objective:**

We sought to determine the effects of oxidative stress on mitochondrial function in the pathophysiology of airway inflammation in ozone-exposed mice and human airway smooth muscle (ASM) cells.

**Methods:**

Mice were exposed to ozone, and lung inflammation, airway hyperresponsiveness (AHR), and mitochondrial function were determined. Human ASM cells were isolated from bronchial biopsy specimens from healthy subjects, smokers, and patients with COPD. Inflammation and mitochondrial function in mice and human ASM cells were measured with and without the presence of the mitochondria-targeted antioxidant MitoQ.

**Results:**

Mice exposed to ozone, a source of oxidative stress, had lung inflammation and AHR associated with mitochondrial dysfunction and reflected by decreased mitochondrial membrane potential (ΔΨm), increased mitochondrial oxidative stress, and reduced mitochondrial complex I, III, and V expression. Reversal of mitochondrial dysfunction by the mitochondria-targeted antioxidant MitoQ reduced inflammation and AHR. ASM cells from patients with COPD have reduced ΔΨm, adenosine triphosphate content, complex expression, basal and maximum respiration levels, and respiratory reserve capacity compared with those from healthy control subjects, whereas mitochondrial reactive oxygen species (ROS) levels were increased. Healthy smokers were intermediate between healthy nonsmokers and patients with COPD. Hydrogen peroxide induced mitochondrial dysfunction in ASM cells from healthy subjects. MitoQ and Tiron inhibited TGF-β–induced ASM cell proliferation and CXCL8 release.

**Conclusions:**

Mitochondrial dysfunction in patients with COPD is associated with excessive mitochondrial ROS levels, which contribute to enhanced inflammation and cell hyperproliferation. Targeting mitochondrial ROS represents a promising therapeutic approach in patients with COPD.

Chronic obstructive pulmonary disease (COPD) is the third most common cause of death worldwide, mainly as a result of cigarette smoking in the developed world.^1^ COPD is characterized by chronic inflammation and development of airflow limitation caused by airway remodeling and destruction of lung tissue.^1^ Tobacco smoke induces local inflammation and enhances oxidative stress in the lung, affecting the oxidant-antioxidant balance, which is thought to be one of the major driving mechanisms in the development of COPD.^2^ Production of reactive oxygen species (ROS) in the mitochondria occurs as a byproduct of oxidative phosphorylation at the mitochondrial electron transport chain.^3^ Normally, ROS are cleared by the actions of antioxidants, such as glutathione, vitamins C and E, and enzymes, including catalases, peroxidases, and superoxide dismutases.^4^ Increased ROS levels or a reduction in antioxidant capacity can influence redox-sensitive cellular processes, including mitochondrial apoptosis.^5^

The primary function of mitochondria is to produce adenosine triphosphate (ATP), although they are also involved in other cellular functions, including redox homeostasis, ROS and nicotinamide adenine dinucleotide phosphate generation, calcium metabolism, and apoptosis.^6^ Mitochondria can also sense danger signals and induce inflammation by activating and controlling the innate immune system.^7^ Indeed, alterations in cellular metabolism have a profound effect on immunology and cell biology, particularly in patients with cancer in whom mitochondrial function is directly regulated by oncogenes and tumor suppressors and might be involved in oncogenic transformation.^8^

Genetic defects in mitochondrial function are associated with diseases, such as Leigh disease and mitochondrial encephalomyopathy, lactic acidosis, and stroke-like syndrome.^9^ These diseases are associated with mitochondrial DNA mutations, which cause defects in respiratory chain function and energy production. Pathogenic mitochondrial DNA mutations are rare (1:10,000) and largely affect children.^9^ In addition, mitochondrial defects have been linked to diseases of aging resulting from exposure to oxidative stress.^10^

Airway wall remodeling in patients with COPD involves tissue repair and epithelial metaplasia, increased mucous metaplasia and submucosal gland hypertrophy, peribronchial fibrosis, and an increase in airway smooth muscle (ASM) mass.^11^ In addition to their contractile properties, ASM cells produce inflammatory mediators thought to drive, at least in part, the airway wall remodeling seen in patients with COPD.^11^ ASM cells from patients with COPD have a distinct phenotype compared with that of cells from healthy subjects.^12^ Specific therapies targeting ASM cells might be essential in treating airway remodeling in these patients.

Ozone is an oxidizing environmental pollutant associated with worsening of symptoms in patients with asthma and COPD^13^ and increased morbidity and mortality rates in patients with cardiovascular and respiratory disorders.^14,15^ Ozone exposure in mice induces airway inflammation, airway hyperresponsiveness (AHR),^16^ and lung destruction similar to that observed in patients with COPD.^17^ Ozone has been hypothesized to initiate intracellular oxidative stress through formation of ozonide and hydrogen peroxide.^18^ In turn, these are able to produce ROS that can alter gene transcription through redox-mediated signaling pathways.^19^ We previously reported that acute and chronic exposure to ozone results in persistent inflammation and AHR, which are in part reversible by treatment with the antioxidant N-acetylcysteine (NAC).^20,21^

Here we show a marked alteration in mitochondrial function associated with increased inflammation and alterations in AHR in an ozone-induced animal model of COPD. In addition, there is abnormal mitochondrial function in ASM cells from patients with COPD, and this is induced by oxidative stress in ASM cells from healthy subjects. Antioxidants that are targeted to decrease mitochondrial oxidative damage specifically led to reduced inflammation and ASM proliferation.

## Methods

A more detailed description of the Methods can be found in this article's Online Repository at www.jacionline.org.

### Subjects

This study was approved by the Ethics Committee of the Royal Brompton & Harefield Hospitals National Health Service Trust. All subjects provided written informed consent. Healthy nonsmokers (n = 15), healthy smokers (n = 10), and patients with COPD (n = 14) were recruited (Table I). In a separate study endobronchial biopsy specimens were obtained from 4 exsmokers with COPD and 4 healthy exsmokers matched for smoking history, age, and sex (Table II).

### Fiberoptic bronchoscopy and culture of ASM cells

Spirometry was performed and lung function was recorded before bronchoscopy, as previously described.^22^ All subjects had bronchial biopsy specimens taken. ASM cells were isolated from bronchial biopsy specimens, as previously described.^23^ ASM cells were cultured in supplemented Dulbecco modified Eagle medium and used at passages 3 and 6. ASM cells were incubated in supplemented serum-free medium for 18 hours before experiments^23^ and used to measure various parameters of mitochondrial function. In other subjects intact mitochondria were isolated from fresh bronchial biopsy specimens, and membrane potential and mitochondrial ROS levels were measured.

### Mouse AHR and bronchoalveolar lavage

Experiments were performed under a Project License from the British Home Office, United Kingdom, under the Animals (Scientific Procedures) Act 1986. Male C57BL/6 mice (6 weeks old; Harlan, Wyton, United Kingdom) were exposed to ozone (model 500 Ozoniser; Sander, Wuppertal, Germany) mixed with air (3-ppm concentration), as previously described.^20,21^ Lung function and AHR were measured and bronchoalveolar lavage (BAL) fluid and tissue collection was performed 24 hours after the last exposure.^20,21^ Resistance/compliance measures were obtained from tracheostomized and ventilated mice by using whole-body plethysmography (EMMS, Hants, United Kingdom), as previously described.^20^ Lung resistance (R_L_) was expressed as the percentage change from baseline R_L_ with nebulized PBS (Sigma, St Louis, Mo). The concentration of acetylcholine required to increase R_L_ by 100% from baseline was calculated (PC_100_), and the concentration of acetylcholine that increased lung resistance by 100% (−logPC_100_) was taken as a measure of AHR.^20,21^ BAL samples and cytospin preparations were obtained and analyzed, as previously described.^20,21^ Keratinocyte-derived cytokine (KC), IL-6, and GM-CSF levels in BAL fluid were measured with commercial ELISA kits (R&D Systems Europe Ltd, Abingdon, United Kingdom).

### Mitochondria isolated from bronchial biopsy specimens and murine lung tissue

Intact mitochondria were isolated with a Mitochondria Isolated Kit for Tissue (Thermo Scientific, Waltham, Mass), exactly as described by the manufacturer.

### RNA isolation and microarrays

RNA isolation and microarrays were performed exactly as previously described.^24,25^ Quantitation and quality assessment of the RNA preparations were performed by using Nanodrop analysis (Nanodrop Technologies, Wilmington, Del) and the Agilent 2100 bioanalyzer and the RNA 6000 LabChip kit (Agilent Technologies, Palo Alto, Calif), respectively. cDNA was run on Mouse Genome 430 array (HT plate format) Affymetrix chips, and gene expression profiles were examined with Ingenuity pathway analysis (Ingenuity Systems, Redwood City, Calif) and Partek Genomics suit 6.6 beta software. Expression of selected genes was confirmed by means of TaqMan (Applied Biosystems, Foster City, Calif) quantitative PCR analysis.

### Mitochondrial respiration

Mitochondrial respiration was determined by measuring oxygen consumption rates (OCRs) in the extracellular space with a Seahorse XF24 Extracellular Flux Analyzer and the XF Cell Mito Stress Test kit (Seahorse Bioscience, North Billerica, Mass), according to the manufacturer's instructions. ASM cells (10^5^ cells per well) were seeded in a 24-well plate and serum starved overnight.

### ATP determination and complex I activity

ATP levels were quantified with a bioluminescence ATP determination assay (Molecular probes, Eugene, Ore). Complex I enzyme activity levels were determined based on oxidation of NADH to NAD+ with a commercial assay (MitoSciences, Eugene, Ore).

### Mitochondrial membrane potential

Mitochondrial membrane potential (ΔΨm) was measured in human ASM cells and isolated mitochondria (human biopsy and mouse lung) by using the cationic dye 5,5′,6,6′-tetrachloro-1,1′,3,3′-tetraethylbenzimidazolylcarbocyanine iodide (JC-1; Invitrogen, Carlsbad, Calif). ASM cells and isolated intact mitochondria were incubated with 3 μmol/L JC-1 for 30 minutes at 37°C and 5% CO_2_. JC-1 monomers emit green fluorescence, but when they enter live mitochondria, they form J-aggregates, which emit red fluorescence. An increase in the ratio between red and green fluorescence is an indicator of ΔΨm.

### Mitochondrial ROS analysis

Mitochondrial ROS activity was measured with MitoSOX Red (Invitrogen), a redox-sensitive fluorescent probe that is selectively targeted to the mitochondria. ASM cells and intact isolated mitochondria were incubated with 5 μmol/L MitoSOX for 30 minutes at 37°C and 5% CO_2_, and red fluorescence was determined at 510/580 nm by using a fluorescence plate reader or by means of flow cytometry.

### Immunoblotting

Cell, tissue, and mitochondrial protein fractions were separated on 10% NuPAGE gels, and proteins were detected, as previously described.^21^ The following antibodies were used: MitoProfile Total OXPHOS Rodent WB Antibody Cocktail and MitoProfile Total OXPHOS Human WB Antibody Cocktail (MitoSciences).

### Statistical analysis

Data from *in vivo* experiments are expressed as means ± SDs. The Kruskal-Wallis test for ANOVA was used for multiple comparisons of different groups. The Mann-Whitney test was subsequently used when results on the Kruskal-Wallis test were significant. Power calculations determined that 6 animals per group have 80% power to detect significant difference in lung parameters. Data from *in vitro* experiments are expressed as means ± SEMs and were analyzed by using 1-way ANOVA for repeated measures, followed by the Dunnett or Bonferroni *post hoc* test. The Mann-Whitney test was used for comparisons between disease groups. Statistical analysis was performed with GraphPad Prism 4 software (GraphPad Software, San Diego, Calif). A *P* value of less than .05 was accepted as statistically significant.

## Results

### Ozone-induced AHR and lung inflammation in mice

**Fig 1:**
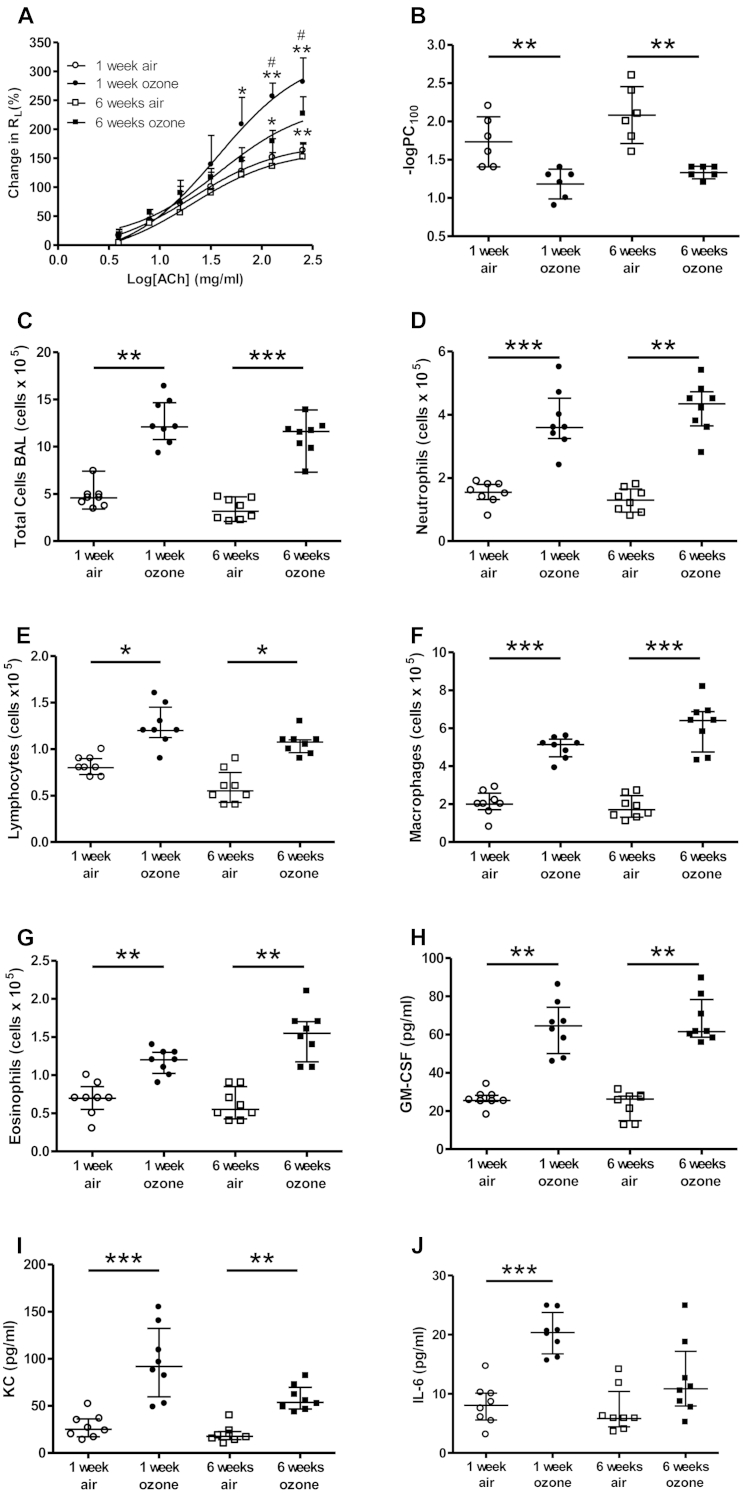
**A** and **B,** Airways hyperresponsiveness measured as R_L_ (Fig 1, *A*) and −logPC_100_ (Fig 1, *B*). **C-J,** BAL fluid total cell (Fig 1, *C*), neutrophil (Fig 1, *D*), lymphocyte (Fig 1, *E*), macrophage (Fig 1, *F*), and eosinophil (Fig 1, *G*) counts and GM-CSF (Fig 1, *H*), KC (Fig 1, *I*), and IL-6 (Fig 1, *J*) levels were measured. Data are expressed as means ± SDs of 6-8 mice per group. *P < .05, **P < .01, and ***P < .001 compared with air-exposed control group. #*P* < .05 compared with 1-week ozone-exposed group.

AHR to acetylcholine was measured in air- and ozone-exposed mice. There were greater increases in R_L_ in mice exposed to ozone for both 1 and 6 weeks compared with that seen in air-exposed animals (Fig 1, *A*). AHR was observed in both 1- and 6-week-exposed mice. The −logPC_100_ value was decreased in 1-week-exposed (1.5-fold) and 6-week-exposed mice (1.6-fold; Fig 1, *B*).

Ozone-exposed mice showed marked increases in total BAL cell counts compared with those in air-exposed control mice after 1 and 6 weeks, with increased neutrophil, lymphocyte, macrophage, and eosinophil counts (Fig 1, *C-G*). One and 6 weeks of ozone exposure also induced an increase in proinflammatory cytokine profiles in BAL fluid, including GM-CSF, KC, and IL-6 (Fig 1, *H-J*). Gene microarray analysis of lung samples from these ozone-exposed mice demonstrated significantly increased (>2-fold) expression of 116 genes (see Table E1 in this article's Online Repository at www.jacionline.org) and reduced (>2-fold) expression of 69 genes (see Table E2 in this article's Online Repository at www.jacionline.org). Gene ontology analysis provided evidence for increased inflammation and cell proliferation at the mRNA level at 6 weeks (Table III). These effects of ozone on inflammation are modulated through oxidative stress.^20^ Gene ontology analysis of the ozone-modulated genes also indicated a significant effect on genes associated with mitochondrial function (Table III).

The top differentially upregulated and downregulated genes in the 6-week ozone model were defined as an ozone gene expression “signature” and compared with the transcriptomic GEO data set (GSE20257) containing gene array data from epithelial cells from smokers and patients with COPD (Global Initiative for Chronic Obstructive Lung Disease [GOLD] stage 1-3). Gene Set Variation Analysis showed increased association of the mouse ozone “signature” with COPD severity (*P* < .05, see Fig E1 in this article's Online Repository at www.jacionline.org).

### Mitochondrial function is impaired in lungs of ozone-exposed mice

**Fig 2:**
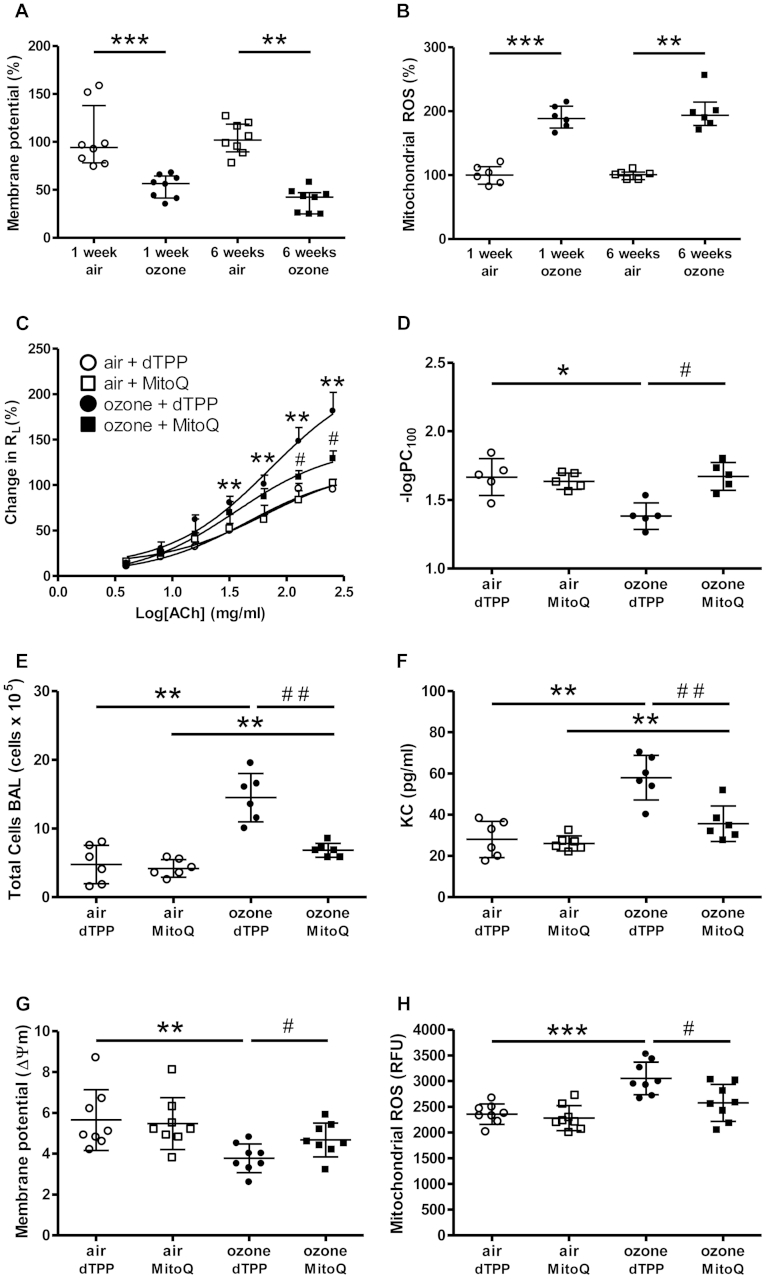
**A, B, G,** and **H,** Membrane potential (ΔΨm; Fig 2, *A* and *G*) and mitochondrial ROS (Fig 2, *B* and *H*) after ozone exposure. **C, D, E,** and **F,** R_L_ (Fig 2, *C*), −logPC_100_ (Fig 2, *D*), BAL total cell counts (Fig 2, *E*), and KC levels (Fig 2, *F*) were determined. ΔΨm (Fig 2, *G*) and ROS content (Fig 2, *H*) were determined in isolated mitochondria. Data are expressed as means ± SDs of 5-8 mice per group. *P < .05, **P < .01, and ***P < .001 compared with air-exposed control group. #*P* < .05 and ##*P* < .01 compared with ozone-exposed group.

We examined whether mitochondrial function was altered in the lungs of ozone-exposed mice. Mitochondria were isolated from lung tissue and incubated with the fluorescent probes JC-1 or MitoSOX. ΔΨm was decreased in 1-week (2.0-fold) and 6-week (2.4-fold) ozone-exposed mice (Fig 2, *A*). Mitochondrial ROS levels were increased in the lungs of 1- and 6-week ozone-exposed mice (1.9- and 2.1-fold, respectively; Fig 2, *B*). ATP content was decreased 2.5- and 1.7-fold in intact mitochondria isolated from 1- and 6-week ozone-exposed mice, respectively (see Fig E2, *A*, in this article's Online Repository at www.jacionline.org). Complex I enzyme activity levels measured based on NADH consumption rates in isolated mitochondria were decreased 1.4- and 4.6-fold in 1- and 6-week ozone-exposed mice, respectively (see Fig E2, *B*).

Protein expression levels measured by using Western blot analysis (see Fig E2, *C*) were reduced for complex I (NADH dehydrogenase), complex III (cytochrome bc_1_ complex), and complex V (FoF1-ATPase; see Fig E2, *D-F*) in 1- and 6-week ozone-exposed groups compared with those in air-exposed control ice. Complex III and V levels were more profoundly decreased in 6-week-exposed compared with 1-week-exposed mice, whereas complex I reduction was similar for both ozone-exposed groups.

### MitoQ reverses ozone-induced AHR and inflammation

MitoQ is a combination of ubiquinone, an endogenous antioxidant and component of the mitochondrial electron transport chain, and a decyltriphenylphosphonium bromide (dTPP) cation, the latter allowing it to concentrate in mitochondria.^26^ Treatment of mice with MitoQ (5 mg/kg administered intraperitoneally) resulted in a significant reversal of ozone-induced AHR compared with treatment with the control cation, dTPP, as measured based on −logPC_100_ values (Fig 2, *C* and *D*). MitoQ treatment also resulted in a reduction in ozone-induced total BAL cell counts (Fig 2, *E*) and BAL KC levels (Fig 2, *F*) compared with dTPP treatment alone. This was associated with a restoration of ΔΨm (Fig 2, *G*), a reduction in ROS levels in isolated mitochondria (Fig 2, *H*), and a reduction in total cellular ROS levels (see Fig E2, *G*).

### ASM cells from patients with COPD demonstrate mitochondrial dysfunction

**Fig 3:**
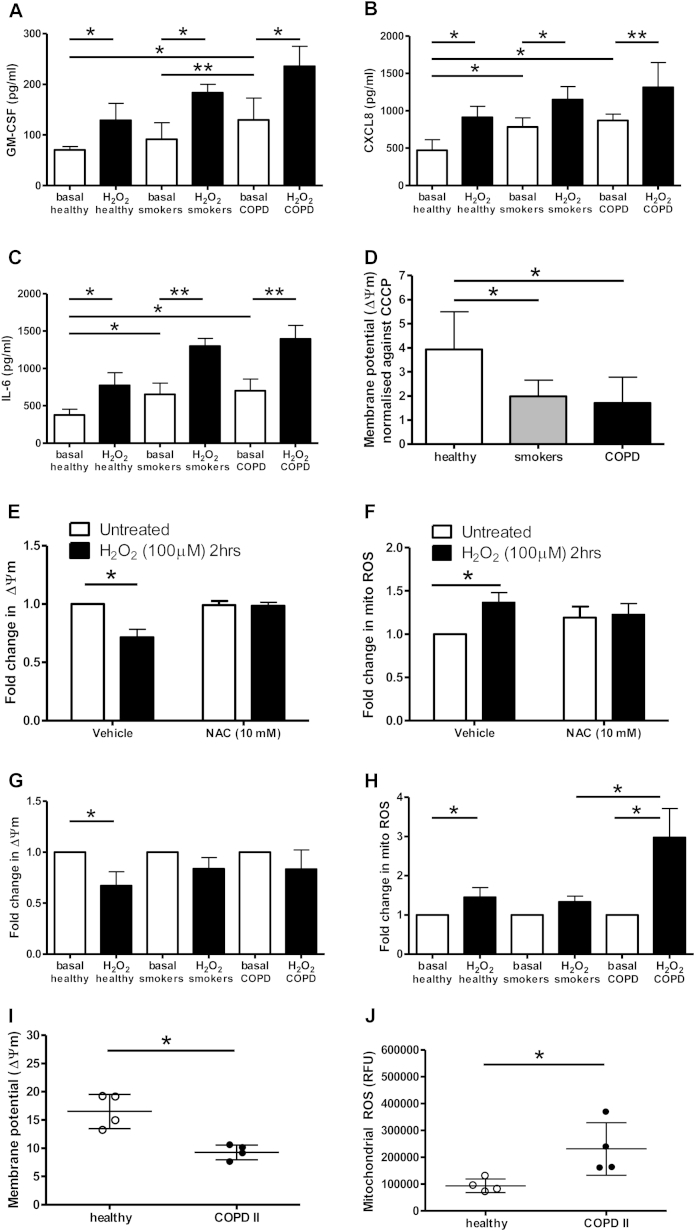
ASM cells from healthy subjects, smokers, and patients with COPD were treated with H_2_O_2_ with and without NAC before treatment. **A-C**, GM-CSF (Fig 3, *A*), CXCL8 (Fig 3, *B*), and IL-6 (Fig 3, *C*) levels were determined by means of ELISA. **D-H**, ΔΨm (Fig 3, *D, E* and *G*) and mitochondrial ROS values (Fig 3, *F* and *H*) were also measured. *Bars* represent means ± SEMs of 5 to 6 donors per group. *P < .05 and **P < .01. **I-J**, Intact mitochondria were isolated from bronchial biopsies from healthy subjects and COPD patients. ΔΨm (Fig 3, *I*) and mitochondrial ROS values (Fig 3, *J*) were measured. Dot plots represent means ± SD of 4 subjects or patients per group.

We studied ASM cells cultured from bronchial biopsy specimens obtained from patients with COPD of GOLD stage 1 or 2 and ASM cells from healthy nonsmokers and healthy smokers for comparison (see Table I for the characteristics of these subjects). Basal GM-CSF (2.5-fold), CXCL8 (1.6-fold), and IL-6 (1.8-fold) secretions from ASM cells isolated from smokers and patients with COPD and studied at passage 4 were increased compared with those from healthy control subjects (Fig 3, *A-C*). IL-6 secretion was increased (2.1-fold) in ASM cells from smokers compared with those from healthy control subjects (Fig 3, *C*). Exposure to H_2_O_2_ (100 μmol/L for 4 hours) increased GM-CSF, CXCL8, and IL-6 secretion from ASM cells in all subject groups (Fig 3, *A-C*).

ΔΨm (Fig 3, *D*) was lower in unstimulated ASM cells from smokers (2.0-fold) and patients with COPD (2.3-fold) compared with those from healthy control subjects. H_2_O_2_ exposure led to a reduction in ΔΨm and an increase in mitochondrial ROS levels, which were reversed by prior exposure to NAC (Fig 3, *E* and *F*). However, H_2_O_2_ did not significantly decrease ΔΨm in ASM cells from smokers and patients with COPD (Fig 3, *G*). H_2_O_2_ increased mitochondrial ROS levels in all groups, with the greatest stimulation in ASM cells isolated from patients with COPD (Fig 3, *H*). Basal mitochondrial ROS levels, as determined by the mean fluorescence intensity, differed between healthy nonsmokers (median, 236.0; 95% CI, 154.3-331; n = 9), healthy smokers (median, 587.9; 95% CI, 130.9-1045; n = 8), and patients with COPD (median, 337.0; 95% CI, 228.2-804.7; n = 16), although this did not reach significance (*P* = .06, ANOVA). Within each patient group, there was no age-related difference in mitochondrial ROS and ΔΨm values.

To determine whether the changes in mitochondrial ROS and membrane potential observed in ASM cells reflect changes in the lung at the organ level, we isolated intact mitochondria from bronchial biopsy specimens from control subjects and patients with COPD. ΔΨm was lower in lungs from patients with COPD compared with those from healthy control subjects (Fig 3, *I*). Mitochondrial ROS levels were increased in patients with COPD compared with those in healthy control subjects (Fig 3, *J*). These results are similar to those observed in ASM cells and confirm the ASM cell phenotype.

**Fig 4:**
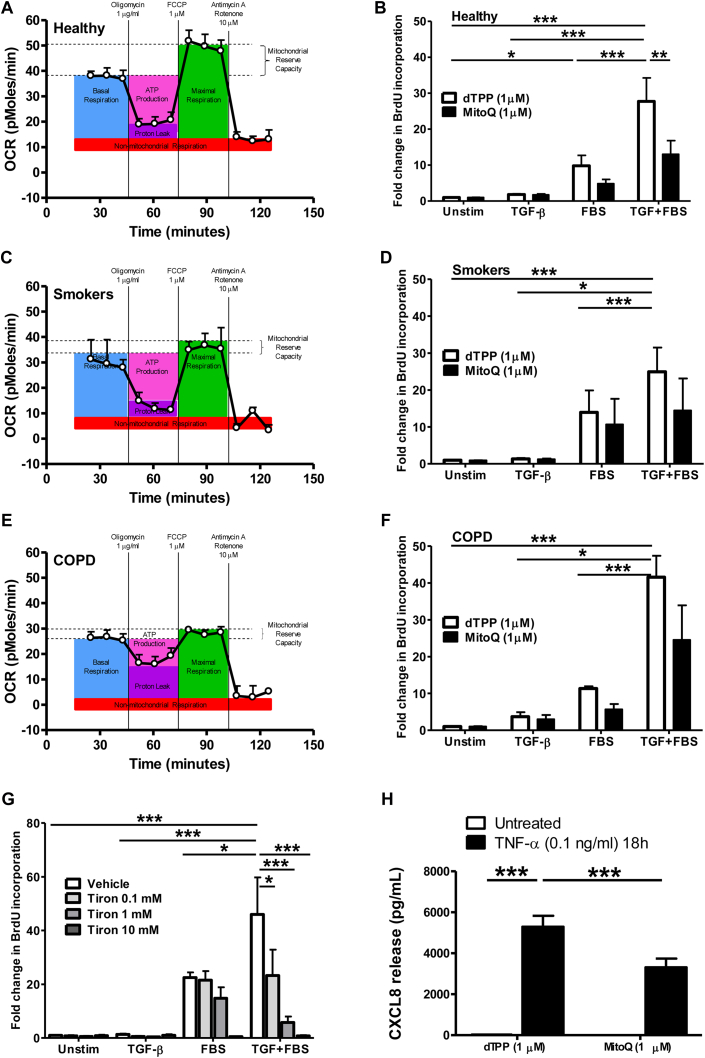
**A-F,** Baseline mitochondrial respiration determined by measuring OCR and the effect of MitoQ on TGF-β ± FBS–induced DNA synthesis were determined in ASM cells from healthy subjects (Fig 4, *A* and *B*), smokers (Fig 4, *C* and *D*), and patients with COPD (Fig 4, *E* and *F*). **G** and **H,** Effects of Tiron on TGF-β ± FBS–induced DNA synthesis (Fig 4, *G*) and MitoQ on TNF-α–induced CXCL8 release (Fig 4, *H*) were determined. *Bars* represent means ± SEMs of 3 (Fig 4, *A*, *C*, *E*, *G*, and *H*), 8 (Fig 4, *B*), 6 (Fig 4, *D*), and 4 (Fig 4, *F*) donors. *P < .05, **P < .01, and ***P < .001.

In view of the differences in mitochondrial ROS and membrane potential observed in endobronchial biopsy specimens and ASM cells from patients with COPD compared with cells from healthy nonsmokers, we next made real-time measurements of the OCR as an indicator of mitochondrial respiration (Fig 4 and see Fig E3 in this article's Online Repository at www.jacionline.org).

A significant reduction in basal OCR was measured in ASM cells isolated from patients with COPD (*P* < .05; Fig 4, *E*, and see Fig E3, *A*) compared with ASM cells isolated from healthy control subjects (Fig 4, *A*), although there was a trend toward a reduction in basal OCRs in healthy smokers (*P* = .0512; Fig 4, *C*, and see Fig E3, *A*). In the presence of oligomycin, which inhibits the activity of ATP synthase, the ATP-linked OCR was measured. Carbonyl cyanide *p*-(trifluormethoxy)phenylhydrazone, an uncoupler of oxidative oxidation, was added to assess maximal respiration capability. This was followed by adding rotenone and antimycin A, which are complex I and III inhibitors, respectively, to determine maximal OCR. The OCR linked to ATP generation (see Fig E3, *B*) was reduced in ASM cells from patients with COPD only. However, the maximal OCR (see Fig E3, *C*) was significantly lower in ASM cells from both smokers and patients with COPD, resulting in a significantly attenuated reserve capacity in mitochondria from smokers and cells from patients with COPD (see Fig E3, *D*).

The membrane potential in unstimulated ASM cells from healthy smokers and nonsmokers could be fully depolarized with the uncoupler carbonyl cyanide *m*-chlorophenyl hydrazone (see Fig E3, *E*). This did not occur with cells from patients with COPD, possibly because these already had a reduced membrane potential. Inhibition of complex III with antimycin A prevents all mitochondrial respiration, leading to enhanced mitochondrial ROS in ASM cells from all subject groups, with the highest increase in ASM cells from healthy subjects (see Fig E3, *F*).

### Oxidative stress reduces OXPHOS expression in ASM cells

There was a reduced production of cellular ATP in cells from healthy smokers and patients with COPD compared with that seen in healthy nonsmokers (see Fig E4, *A*, in this article's Online Repository at www.jacionline.org), and H_2_O_2_ exposure reduced mitochondrial ATP production in ASM cells from healthy smokers and nonsmokers but not in patients with COPD. This was associated with a reduction in expression of mitochondrial complex proteins (see Fig E4, *B-D*). Complex I and V expression levels were lower in ASM cells of smokers and patients with COPD at baseline (see Fig E4, *B* and *D*), and complex III levels were reduced in ASM cells from patients with COPD only (see Fig E4, *C*).

### Inhibition of mitochondrial function attenuates ASM proliferation and inflammatory responses

TNF-α significantly enhanced CXCL8 release from cells, and this was significantly reduced by MitoQ compared with the effect seen with its control, dTPP (Fig 4, *H*). ASM cells from healthy subjects (Fig 4, *B*) demonstrated less proliferation in response to TGF-β and FBS than cells from smokers (Fig 4, *D*) and patients with COPD (Fig 4, *F*). MitoQ also significantly reduced the proliferation seen in ASM cells from healthy subjects and patients with COPD by 50% compared with dTPP. The more potent mitochondrial ROS scavenger Tiron (Acros Organics, Geel, Belgium) prevented TGF-β– and FBS-induced ASM proliferation to a greater extent (Fig 4, *G*). No treatment used resulted in significant levels of cell death (data not shown).

## Discussion

We report decreased ΔΨm, ATP content, and lower complex protein expression and activity levels in the lungs of mice chronically exposed to the oxidant gas ozone. In addition, these ozone-induced processes were reversed by inhibition of mitochondrial ROS with the mitochondria-targeted antioxidant MitoQ, which was accompanied by reversal of ozone-induced lung inflammation and AHR. In addition, we demonstrate a profound mitochondrial dysfunction of ASM cells obtained from smokers and patients with COPD when compared with ASM cells from healthy subjects. This is supported by the observations that ΔΨm, ATP content, respiratory complex protein expression, and activity levels were all decreased. Thus ASM cells from patients with COPD are unable to provide adequate respiration and had a severely reduced respiratory reserve capacity.

Targeting mitochondrial ROS with the mitochondria-targeted antioxidant MitoQ and the mitochondria-localized antioxidant Tiron led to a reduction of the increased cytokine secretion and proliferation of ASM cells from patients with COPD, highlighting the role for excessive mitochondrial ROS in driving these abnormalities of the ASM in patients with COPD. In addition, intact mitochondria isolated from endobronchial biopsy specimens of healthy subjects and patients with COPD showed a similar difference in phenotype as seen in cultured ASM cells with increased mitochondrial ROS content and decreased ΔΨm levels in mitochondria from patients with COPD compared with healthy control subjects. This also highlights the ability of isolated ASM cells from patients with COPD to maintain the COPD phenotype even through several passages in culture. The intermediate mitochondrial phenotype seen in healthy smokers compared with patients with COPD and healthy nonsmokers also indicates that these differences are related to disease rather than smoke *per se*. Follow-up of these patients over several years to determine whether they succumb to COPD will be of interest in future studies.

The airways in patients with COPD are remodeled with a thickened epithelium, lamina propria, and adventitia, with ASM hyperplasia and hypertrophy and deposition of collagen that might all contribute to the development of AHR.^27,28^ In the ozone model AHR is associated with the presence of increased isometric contractile responsiveness of the airways to acetylcholine,^29^ which was due to an increased activation of the p38 mitogen-activated protein kinase. Therefore the AHR we observed here was likely to be secondary to direct changes in the ASM. Both ozone-induced AHR and inflammation were reversed by MitoQ, suggesting that excessive mitochondrial ROS might contribute directly to these effects. MitoQ has been shown to increase mitochondrial matrix calcium levels, which could favor Ca^2+^ overload^30^ and might directly interfere with the contractile response of ASM. Our data also suggest that targeting mitochondrial ROS with mitochondria-directed antioxidants might be more effective than extracellular antioxidants, such as NAC.^20^

In a recent study short-term cigarette smoke exposure of mice resulted in changes in lung energy metabolism, with redirection of glucose metabolism toward the pentose phosphate pathway,^31^ which resulted in a lower substrate supply (pyruvate) to the mitochondria that can directly influence mitochondrial function. However, there was increased expression of complexes II, III, and IV and ATPase (complex V) in this model that might be viewed as a compensatory response to the limited substrate (pyruvate) supply to mitochondria. It is likely that the oxidative drive provided by ozone exposure is much greater than that with cigarette smoke. Indeed, this is the main reason why the ozone exposure model is able to induce a COPD-like phenotype consisting of chronic inflammation, cell infiltration, steroid resistance, emphysema, and lung remodeling in a matter of weeks instead of months, which is more common in cigarette smoke mouse models. In addition, the gene expression signature in the ozone model associates with human COPD severity. This association was not present in a short-term cigarette smoke model when compared with the same human data set (data not shown). This highlights that although in both models oxidative stress drives the induced phenotype, ozone exposure seems to induce a faster and stable COPD phenotype compared with cigarette smoke exposure.

Little is known currently about the functional state of mitochondria in patients with COPD. A recent study examined mitochondrial structure in airway epithelial cells of patients with advanced COPD (GOLD stage 4). Hoffmann et al^32^ showed that bronchial epithelial cells from exsmokers with COPD showed damaged mitochondrial structure, with depletion of cristae, increased branching, elongation, and swelling of the mitochondria. This was accompanied by increased manganese superoxide dismutase and peroxisome proliferator-activated receptor γ coactivator 1α expression and also augmented levels of the complex VF1α subunit levels. Although the respiratory capacity of these cells was not measured, the expression of these mitochondrial proteins would suggest that epithelial cells, despite expressing damaged mitochondria, might be coping well with oxidative stress. Mitochondrial dysfunction has also been reported in skeletal and respiratory muscles of patients with COPD,^33,34^ although these changes might reflect altered muscle physiology in sedentary patients rather than reflecting differences in patients with COPD within the lung.

Abnormalities in mitochondrial function have been described in ASM cells from asthmatic patients. Increased mitochondrial mass and enhanced mitochondrial biogenesis were linked to increased ASM proliferation in asthmatic patients.^35^ This would suggest that the mitochondria of ASM cells from asthmatic patients were able to provide adequate respiration in the context of mitochondrial respiration at an increased level. ASM cells from asthmatic patients, particularly those with severe asthma, have a defective Nrf2–antioxidant response element binding in addition to a reduced expression of the antioxidant hemoxygenase-1.^36^ Decreased ATP levels and mitochondrial abnormalities, including decreased cytochrome c oxidase activity, the loss of cristae, and mitochondrial swelling, have been described in the lungs of sensitized mice challenged with ovalbumin.^37^ Compounds that act as oxidant scavengers, such as esculetin, vitamin E, and simvastatin, attenuated these features through reversing mitochondrial dysfunction,^38,39^ indicating that chronic mitochondrial ROS might be detrimental to mitochondrial and airway function in asthma models.

COPD can be considered a disease of the aging lung.^40^ Mitochondria do more than just supply cellular energy needs to survive. The cell has evolved to provide numerous resources and metabolic mechanisms to support the mitochondrial compartment.^10^ Consequently, mitochondria participate in a myriad of cellular processes by regulating and sensing various homeostatic mechanisms that in turn play a vital role in aging and in regulating the cell's response to particular stresses.^10^ There might be additional functional consequences of this acquired mitochondrial dysfunction in cells from patients with COPD, such as alterations in antiviral signaling^41^ and susceptibility to cancer.^8^

We have previously reported increased oxidative and nitrosative stress in patients with COPD, with the expression of nitrotyrosine-positive cells inversely correlated with lung function.^42^ The enhanced production of oxidative stress by mitochondria from patients with COPD might contribute to this increased nitrotyrosine expression because of interactions with nitric oxide (NO), levels of which are increased in patients with COPD because of increased expression of NO-synthesizing enzymes.^42^ Reactive nitrogen species, formed through the reaction of NO with superoxide anions, have potent proinflammatory and oxidizing actions.^43^ NO also modulates the expression of cytochrome c oxidase.^44^ Cytochrome c oxidase (complex IV of the mitochondrial electron transport chain) is the primary site of cellular oxygen consumption and is central to oxidative phosphorylation and ATP generation.^44^

Under normal physiologic conditions, oxidants serve as “redox messengers” regulating intracellular signaling, whereas excess oxidants can induce irreversible damage to cellular components, leading to apoptosis and/or altered cellular function, including innate immunity through mitochondria.^45^

Our observation of mitochondrial dysfunction in patients with COPD is based on experiments on the ozone-exposed mouse model and on ASM cells obtained from a restricted group of patients with COPD. The ozone-exposed mouse model is driven by oxidative stress, although recapitulating the inflammation and emphysema aspects of COPD might not mimic all its pathophysiologic features, particularly small airway disease in view of its limited branching anatomy compared with human subjects. ASM cells were studied at passage 4 or 5 in culture and might not have kept their original phenotypic abnormalities on successive passages despite expressing smooth muscle actin and myosin. However, ASM cells cultured from asthmatic patients show abnormal levels of mitochondrial fragmentation.^46^ Finally, we have restricted ourselves to patients with moderate-to-severe COPD when this is a complex disease that can manifest in more diverse phenotypes. Future studies of mitochondrial dysfunction in patients with COPD should examine fresh tissues to confirm these abnormalities in diverse phenotypes, including frequent exacerbators and those with emphysema. In addition, other cells can be examined, such as bronchial epithelial cells, in which mitochondrial abnormalities have been recently reported.^32,47^

In summary, we have shown mitochondrial dysfunction and excessive mitochondrial ROS in ASM cells of patients with COPD, and this might contribute to their proinflammatory and enhanced proliferative properties. In addition, this acquired mitochondrial dysfunction might be important in the development of comorbidities and in responses to infections that are common in patients with COPD. Mitochondria-directed antioxidants represent a more logical approach to the treatment of COPD by reversing inflammatory and remodeling processes in patients with COPD that are not treatable at present.

**Key messages**

• Ozone exposure in mice induces inflammation, airway hypersensitivity, and mitochondrial dysfunction, which are reversed by treatment with the mitochondria-specific antioxidant MitoQ.
• ASM cells isolated from patients with COPD have impaired mitochondrial function, which is not further induced by oxidative stress.
• Oxidative stress induces mitochondrial dysfunction in ASM cells from healthy subjects compared with ASM from patients with COPD, which can be prevented by mitochondria-directed antioxidant treatment.

## Figures and Tables

**Table I:** Demographics of subjects undergoing bronchoscopy from whom ASM cells were obtained

	Healthy subjects	Smokers	Patients with COPD
No.	15	10	14
Age (y)	41.20 ± 3.74	57.10 ± 1.85∗	68.2 ± 1.85‡‖
Sex (male/female)	9/6	7/3	7/7
Pack years smoking	NA	34.67 ± 3.64	50.95 ± 4.68§
FEV_1_ (L)	4.10 ± 0.21	2.85 ± 0.23†	1.69 ± 0.12‡§
FEV_1_ (% predicted)	106.70 ± 3.1	92.75 ± 5.76	61.66 ± 3.72‡¶
FVC (L)	5.16 ± 0.39	3.96 ± 0.33	3.23 ± 0.22†
FVC (% predicted)	111.2 ± 4.31	105.7 ± 5.73	101.4 ± 5.22
FEV_1_/FVC ratio (%)	88.33 ± 3.04	75.40 ± 3.33	52.57 ± 3.02‡¶

*FVC*, Forced vital capacity; *NA*, not applicable.

∗ *P* < .05 versus healthy subjects.

† *P* < .01 versus healthy subjects.

‡ *P* < .001 versus healthy subjects.

§ *P* < .05 versus smokers.

‖ *P* < .01 versus smokers.

¶ *P* < .001 versus smokers.

**Table II:** Demographics of subjects undergoing bronchoscopy from whom mitochondria were freshly isolated

	Healthy subjects	Patients with COPD
No.	4	4
Smoking (pack years)	34 ± 6	36 ± 8
FEV_1_ (% predicted)	99 ± 6	72 ± 2∗

∗ *P* < .01 versus healthy subjects.

**Table III:** Gene ontology analysis of ozone-treated lungs: Identification of processes

	*P* value	Benjamini	FDR
Processes upregulated			
Cyclin/proliferation	5.4E-06	9.8E-04	1.0E-03
Inflammation	1.7E-02	1.0E-03	2.6E-02
Lipid biosynthesis	1.1E-04	2.0E-03	4.7E-02
Processes downregulated			
Mitochondria function	1.3E-07	1.9E-06	8.7E-05

*FDR*, False discovery rate.

## Supplementary data

Online Repository Data

Table E1

Table E2

Fig E1

Fig E2

Fig E3

Fig E4
