# Supplementary material for: Oxidative stress–induced mitochondrial dysfunction drives inflammation and airway smooth muscle remodeling in patients with chronic obstructive pulmonary disease
Source: J Allergy Clin Immunol. 2015 Sep;136(3):769–80. doi: 10.1016/j.jaci.2015.01.046 (PMC4559140; doi:10.1016/j.jaci.2015.01.046)
Supplement: Online Repository Data [file mmc1.docx]

**Supplementary methods and results**

**Oxidative stress-induced mitochondrial dysfunction drives inflammation and airway smooth muscle remodelling in COPD**

Coen H. Wiegman, PhD*^†^, Charalambos Michaeloudes. PhD^†^, Gulammehdi Haji, MD^†^, Priyanka Narang, PhD, Colin J. Clarke, MSc, Kirsty E. Russell, PhD, Wuping Bao, MD, Stelios Pavlidis, PhD^^^, Peter J. Barnes, PhD, Justin Kanerva, MSc^#^, Anton Bittner, PhD^#^, Navin Rao, PhD^#^, Michael P. Murphy, PhD^‡^, Paul A. Kirkham, PhD, Kian Fan Chung, MD, Ian M. Adcock, PhD, on behalf of COPDMAP.

Airway Disease Section, National Heart & Lung Institute, Imperial College London, London SW3 6LY, UK. ^^^Janssen Research & Development, High Wycombe, HP12 4DP, UK. ^#^Janssen Research & Development LLC, San Diego, CA 92121, USA. ^‡^MRC Mitochondrial Biology Unit, Cambridge, CB2 0XY, UK.

^†^These authors contributed equally to this work.

**Methods**

**Subjects**

This study was approved by the Ethics Committee of the Royal Brompton & Harefield Hospitals National Health Service Trust. All subjects gave written informed consent. Healthy non-smokers, healthy smokers and COPD patients were recruited (Table I). Healthy non-smokers (n=15), healthy smokers (n=10) and COPD patients (n=14) were recruited (Table 1). In a separate study endobronchial biopsies were obtained from 4 ex-smoker COPD patients and 4 healthy ex-smokers matched for smoking history, age and sex (Table 2).

**Fibreoptic bronchoscopy, collection and processing of bronchial biopsies**

Spirometry was performed and lung function recorded before bronchoscopy (1). Volunteers attended the bronchoscopy suite at 08.30h after overnight fasting and were pre-treated with atropine (0.6mg *i.v.*) and midazolam (5–10mg *i.v.*). Oxygen (O_2_) (3L·min^–1^) was administered *via* nasal prongs and O_2_ saturation was monitored with a digital oximeter. A fibreoptic bronchoscope (Olympus BF10 Key-Med, Southend, UK) was passed through the nasal passages into the trachea and four bronchial mucosal biopsy specimens were taken from segmental and subsegmental airways of the right lower and upper lobes using cupped forceps.

**Airway Smooth Muscle (ASM) isolation and culture**

ASM cells were isolated from bronchial biopsies as previously described (2). Briefly, biopsies were cut into small pieces (<1mm^2^) and placed in DMEM supplemented with 4mM L-glutamine, 20U/l penicillin, 20μg/ml streptomycin, 2.5μg/ml amphotericin B and 10% FBS, at 37°C and 5% CO_2_. Cells between passages 3 and 6 were used for the experiments. ASM cells were incubated in serum-free medium, containing phenol-free DMEM supplemented with 1mM sodium pyruvate, 4mM L-glutamine, 1:100 non-essential amino acids, 0.1% BSA, 20U/l penicillin, and 20μg/ml streptomycin and 2.5μg/ml amphotericin B for 18hrs before experiments.

**Mice**

Experiments were performed under a Project License from the British Home Office, UK, under the Animals (Scientific Procedures) Act 1986. Male C57BL/6 mice (6 weeks old, Harlan, Wyton, UK), were housed in groups of 5 for the duration of the experiment. Mice were exposed to ozone (model 500 Ozoniser, Sander, Wuppertal, Germany) mixed with air (3ppm concentration). Mice were exposed for 3 hours a day, 2 times a week for a period of 1 or 6 weeks. Control groups were exposed to normal air. Twenty-four hours after the last exposure, mice were anaesthetised and prepared for lung function analysis and airway hyperresponsiveness (AHR) measurements and BAL and tissue collection (3, 4).

**Measurement of airways hyperresponsiveness (AHR)**

Tracheostomised mice were ventilated (MiniVent type 845, Hugo Sach Electronic, Germany) at 250 breaths/minute and with a tidal volume of 250μl. Mice were continuously monitored in a whole body plethysmograph with a pneumotachograph connected to a transducer (EMMS, Hants, UK). Transpulmonary pressure was assessed via an oesophageal catheter (EMMS). Pulmonary resistance (R_L_) was recorded for 3 minute periods during increasing concentrations (4-256mg/ml) of acetylcholine (Sigma) administered with an Aeroneb^®^ Lab Micropump Nebulizer (EMMS). R_L_ was expressed as percentage change from baseline R_L_ with nebulised PBS (Sigma). The concentration of acetylcholine required to increase R_L_ by 100% from baseline was calculated (PC_100_) and -log PC_100_ taken as a measure of AHR (3, 4).

**Bronchoalveolar lavage**

Bronchoalveolar lavage (BAL) samples were obtained as previously described (3, 4). Lungs were rinsed three times with 0.8ml cold PBS via an endotracheal tube and retrieved as BAL fluid. BAL samples were centrifuged to isolate cells and cell pellets resuspended in 200μl PBS and counted on a haemocytometer (total cell count). BAL fluid was frozen in -80°C until analysis. BAL cell samples were centrifuged onto glass slides by cytospin centrifugation (30xg, 6mins, Shandon Cytospin 4; Thermo Electron Corporation, Waltham, MA, US). Cells on glass slides were stained using Diff-Quick kit (Reagena, Toivala, Finland) and counted under an optical microscope (Olympus BH2, Olympus Optical Company Ltd., Tokyo, Japan). KC, IL-6 and GM-CSF in BAL supernatants were measured using commercial ELISA kits (R&D Systems Europe Ltd, Abingdon, UK).

**RNA isolation and Microarrays**

This was performed as previously described (5, 6). Total RNA was extracted from frozen lung tissue using TRIzol reagent (Invitrogen, Paisley, UK) followed by mirVANA column isolation (Ambion, TX, USA). DNA was removed using on column DNase (Qiagen, UK). Quantitation and quality assessment of the RNA preparations were performed by Nanodrop analysis (Nanodrop Technologies, Wilmington, DE) and Agilent 2100 bioanalyzer and the RNA 6000 LabChip kit (Agilent Technologies, Palo Alto, CA), respectively. Probes were generated in one batch. Initial amplification was carried out using NuGEN V2 amplification, following manufacturer’s instructions. The cDNA was purified, quantified and integrity measured. Finally 1.875μg of cDNA progressed to enzymic and chemical fragmentation, biotin end labelling and hybridization to Mouse Genome 430 array (HT plate format) Affymetrix chips. Gene expression profiles were examined using Ingenuity pathway analysis (Ingenuity Systems, USA) and Partek Genomics suit 6.6 beta. The expression of selected genes was confirmed by TaqMan (Applied Biosystems) qPCR analysis.

**Mitochondrial Respiration**

Mitochondrial respiration was determined by measuring the oxygen consumption rate (OCR) in the extracellular space by a Seahorse XF24 Extracellular Flux Analyzer using the XF Cell Mito Stress Test kit (Seahorse Bioscience, Massachusetts, US) according to manufacturer’s instructions. ASM cells (10^5^ cells/well) were seeded in a 24-well plate and were serum-starved overnight. Basal respiration was determined before sequentially treating with oligomycin (1µg/mL), carbonyl cyanide-p-trifluoremethoxyphenylhydrozone (FCCP; 1µM), rotenone (10µM) and antimycin A (10µM) to manipulate mitochondrial function. Oligomycin inhibits ATP synthase (Complex V) to reveal the proportion of basal OCR linked to ATP generation. FCCP uncouples the electron transport chain from ATP synthase to reveal the maximal OCR. Rotenone inhibits complex I and antimycin A inhibits complex III to determine non-mitochondrial OCR.

**Mitochondria isolation**

Intact mitochondria were isolated using a Mitochondria Isolation Kit for Tissue (Thermo Scientific). Briefly, 20mg of lung tissue was homogenised by Dounce homogenisation. Tissue was washed with PBS with added protease inhibitors (EDTA free, Roche). Tissue was homogenised with 5 strokes of Dounce A and cells were subsequently ruptured with 20 strokes of Dounce B (Dounce tissue grinder set; Sigma). Two differential centrifugation steps were performed to isolate organelle fractions. A large organelle fraction (e.g. plasma membrane, ER, Golgi) was isolated after centrifugation at 700xg for 10 minutes. A second faster spin at 12,000xg for 5 minutes pelleted the mitochondrial fraction. The supernatant was collected as cytosolic fraction. All fractions were frozen at -80°C until analysis.

**Immunoblotting**

Mitochondrial fractions (15μg protein) were separated on 10% NuPAGE gels and transferred to nitrocellulose using iBlotTM (Invitrogen, Paisley, UK). Membranes were blocked for 1hr at room temperature or overnight at 4°C with 5% milk in Tris-Buffered Saline containing Tween20 (TBS-T; Sigma), prior to probing with primary antibodies. Protein detection was performed with Luminata Western HRP Substrate solutions (Merck Millipore) and densitometry was performed using a Gel-Doc-It imaging system (Photon Imaging Systems, Swindon, UK) (4).

**Antibodies**

MitoProfile® Total OXPHOS Rodent WB Antibody Cocktail and MitoProfile® Total OXPHOS Human WB Antibody Cocktail (MitoScience, Eugene, OR, USA) were used on nitrocellulose membranes. Secondary HRP-linked polyclonal immunoglobulins were obtained from Dako (Glostrup, Denmark).

**Mitochondrial membrane potential (ΔΨm)**

ΔΨm was measured in human ASM cells and isolated intact mitochondria using the cationic dye 5,5’,6,6’-tetrachloro-1,1’,3,3’-tetraethylbenzimidazolylcarbocyanine iodide (JC-1; Invitrogen). JC-1 monomers emit green fluorescence but when they enter live mitochondria they form J-aggregates which emit red fluorescence. Live cells or isolated mitochondria were incubated with JC-1 (3µM) for 30 minutes, at 37°C and 5% CO_2_. Monomeric JC-1 (green fluorescence) was detected at excitation/emission ratios of 485/535nm and J-aggregates (red fluorescence) at 560/595nm, using a fluorescence plate reader (Synergy HT Biotek, Winooski, VT, USA). Alternatively, fluorescence was determined using a flow cytometer (BD Biosciences, Oxford, UK). The ratio between the red and green fluorescence is a direct measure of ΔΨm.

**Mitochondrial ROS analysis.**

Mitochondrial ROS activity was measured with MitoSOX^TM^ Red (Invitrogen), a redox-sensitive fluorescent probe that is selectively targeted to the mitochondria. ASM cells and isolated mitochondria from mouse lungs were incubated with 5μM MitoSOX for 30 minutes at 37°C and 5% CO_2_ and red fluorescence was determined at 510/580nm using a fluorescence plate reader or by flow cytometry.

**ATP determination**

ATP levels were quantified with a bioluminescence ATP determination assay (Molecular probes, Eugene, US).

**Complex expression and activity analysis**

Mitochondrial complex I–V expression levels were determined by Western blotting. Complex I activity levels were determined by the oxidation NADH to NAD^+^ using a commercial assay (Mitoscience).

**Statistical Analysis**

Data from *in vivo* experiments are expressed as mean ± SD. Kruskal-Wallis test for ANOVA was used for multiple comparisons of different groups. Mann-Whitney test was subsequently used when Kruskal-Wallis was significant. Power calculations determined that 6 animals per group have 80% power to detect a significant difference in lung parameters. Data from *in vitro* experiments are expressed as mean ± SEM and were analysed using one-way ANOVA for repeated measures, followed by Dunnet or Bonferroni post-hoc test. Mann-Whitney test was used for comparisons between disease groups. Statistical analysis was performed using the GraphPad Prism 4 software (Prism, San Diego, CA, USA). A P value of <0.05 was accepted as statistically significant.

**References**

1. Chang PJ, Bhavsar PK, Michaeloudes C, Khorasani N, Chung KF. Corticosteroid insensitivity of chemokine expression in airway smooth muscle of patients with severe asthma. J Allergy Clin Immunol 2012, 130:877-85 e5.
2. Michaeloudes C, Sukkar MB, Khorasani NM, Bhavsar PK, Chung KF. TGF-β regulates Nox4, MnSOD and catalase expression, and IL-6 release in airway smooth muscle cells. Am J Physiol - Lung Cell Mol Physiol 2011, 300:L295-L304.
3. Li F, Wiegman CH, Seiffert J, Zhu J, Clarke CJ, Chang Y, et al. Effects of N-acetylcysteine in ozone-induced model of chronic obstructive pukmonary disease. PloS ONE 2013, 8:e80782.
4. Wiegman CH, Li F, Clarke CJ, Jazrawi E, Kirkham P, Barnes PJ, et al. A comprehensive analysis of oxidative stress in the ozone-induced lung inflammation mouse model. Clin Sci (Lond) 2013, 126:425-40.
5. Williams AS, Issa R, Leung SY, Nath P, Ferguson GD, Bennett BL, et al. Attenuation of Ozone-Induced Airway Inflammation and Hyper-Responsiveness by c-Jun NH2 Terminal Kinase Inhibitor SP600125. J Pharmacol Exp Therapeutics 2007, 322:351-9.
6. Perry MM, Baker JE, Gibeon DS, Adcock IM, Chung KF. Airway smooth muscle hyperproliferation is regulated by microRNA-221 in severe asthma. Am J Respir Cell Mol Biol 2014, 50:7-17.

**Supplementary Figure legends**

**Figure E1.** Gene Set Variation Analysis (GSVA) between the top differentially up- and down-regulated genes in the 6 week ozone model (ozone “signature”) and the transcriptomic GEO dataset (GSE20257) containing gene array data from epithelial cells from smokers and COPD patients (GOLD Stage 1, 2 and 3).

**Figure E2.** Mitochondrial dysfunction in ozone-exposed mice. Intact mitochondria were isolated from mouse lung and ATP (A) and Complex protein I activity levels (B) determined. Bars represent mean ± SD of n=6 mice per group. Complex protein expression was assessed by Western Blotting in isolated mitochondria (15μg) (C). Complex proteins I (D), III (E), and V (F) were expressed and bands were analysed by densitometry. Bars represent results of 3 Western blots and are shown as mean ± SD. Cellular ROS levels were determined by MitoSOX incubation in 6-week ozone-exposed mice treated with MitoQ (G). *P<0.05 and **P<0.01. #P<0.05 compared to ozone exposure.

**Figure E3.** Respiration rates and mitochondrial function in ASM cells. ASM cells from healthy subjects, healthy smokers and patients with COPD were grown to confluence and mitochondrial respiration was analysed by measuring oxygen consumption rate (OCR) in the extracellular space using a Seahorse XF24 Extracellular Flux Analyzer. After determination of baseline OCR (A), cells were sequentially treated with oligomycin (1µg/mL), FCCP (1µM), rotenone (10µM), and antimycin A (10µM) in order to determine ATP-linked OCR (B), maximal respiration (C), and reserve capacity (D). ASM cells from healthy subjects, smokers and patients with COPD were treated with CCCP (50µM) for 5mins and changes in mitochondrial membrane potential (ΔΨm) were determined by JC-1 staining (E). Alternatively, cells were treated with antimycin A (10µM) for 30mins and mitochondrial ROS levels were determined by MitoSOX staining (F). Bars represent mean ± SEM of 5 ASM cell donors (Healthy), 6 ASM cell donors (Smokers) and 5-6 ASM cell donors (COPD). *P<0.05 and **P<0.01.

**Figure E4.** Oxidative stress reduces OXPHOS expression and attenuates proliferation and inflammatory responses in ASM cells. ASM cells from healthy subjects, smokers and patients with COPD were treated with H_2_O_2_ (100µM) for 2hrs and ATP levels were determined in whole cell extracts (A). Baseline expression of mitochondrial complex I (B), complex III (C) and complex V (D) protein levels in whole cell extracts were determined by Western blotting. Bars represent mean ± SEM of 5 ASM cell donors from each group.
