# Supplementary material for: Oxidative stress–induced mitochondrial dysfunction drives inflammation and airway smooth muscle remodeling in patients with chronic obstructive pulmonary disease
Source: J Allergy Clin Immunol. 2015 Sep;136(3):769–80. doi: 10.1016/j.jaci.2015.01.046 (PMC4559140; doi:10.1016/j.jaci.2015.01.046)
Supplement: Table E1 [file mmc2.docx]

**Table E1. Differentially expressed genes in ozone-treated lungs; upregulated genes.**

| **Gene Symbol** | **Entrez Gene** | **Ozone vs Air** | **Ozone vs Air** | **Ozone vs Air** |
| --- | --- | --- | --- | --- |
|  |  | **Fold Change** | **P Value** | **FDR** |
| Sprr1a | 20753 | 13.79 | 2.2E-12 | 1.1E-08 |
| Ereg | 13874 | 9.65 | 1.2E-13 | 1.1E-09 |
| Orm1 | 18405 | 7.60 | 8.0E-11 | 2.1E-07 |
| Plunc | 18843 | 6.81 | 1.0E-03 | 2.8E-02 |
| Orm2 | 18406 | 6.31 | 2.1E-10 | 4.6E-07 |
| Gp2 | 67133 | 5.85 | 7.9E-06 | 1.0E-03 |
| Saa3 | 20210 | 5.83 | 5.2E-07 | 1.0E-04 |
| S100a14 | 66166 | 5.63 | 2.9E-17 | 1.3E-12 |
| Agr2 | 23795 | 5.54 | 4.4E-05 | 3.3E-03 |
| Ltf | 17002 | 5.25 | 2.6E-06 | 4.0E-04 |
| Sprr2a1 | 100042514 | 4.31 | 6.2E-11 | 1.7E-07 |
| Lcn2 | 16819 | 4.16 | 5.0E-10 | 9.0E-07 |
| Ccna1 | 12427 | 4.13 | 1.5E-05 | 1.5E-03 |
| Cep55 | 74107 | 3.66 | 2.7E-06 | 5.0E-04 |
| Hpx | 15458 | 3.47 | 1.2E-08 | 8.4E-06 |
| Cdk1 | 12534 | 3.42 | 1.0E-07 | 4.1E-05 |
| Psapl1 | 76943 | 3.42 | 2.3E-08 | 1.3E-05 |
| Pbk | 52033 | 3.28 | 4.2E-08 | 2.1E-05 |
| E2f7 | 52679 | 3.14 | 9.8E-07 | 2.0E-04 |
| Ect2 | 13605 | 3.11 | 3.4E-09 | 3.6E-06 |
| Atp10b | 319767 | 3.04 | 1.0E-07 | 4.1E-05 |
| Fst | 14313 | 3.04 | 1.0E-04 | 8.0E-03 |
| Prc1 | 233406 | 3.02 | 5.1E-07 | 1.0E-04 |
| Spag5 | 54141 | 2.98 | 2.0E-05 | 1.9E-03 |
| Ccno | 218630 | 2.97 | 1.4E-05 | 1.4E-03 |
| Top2a | 21973 | 2.87 | 1.6E-06 | 3.0E-04 |
| Psat1 | 107272 | 2.85 | 6.9E-10 | 1.1E-06 |
| Slpi | 20568 | 2.83 | 6.4E-06 | 8.0E-04 |
| Fignl1 | 60530 | 2.83 | 1.3E-10 | 3.3E-07 |
| Oit1 | 18300 | 2.81 | 1.9E-03 | 4.0E-02 |
| Ccnb1 | 268697 | 2.79 | 1.0E-04 | 6.6E-03 |
| A930038C07Rik | 68169 | 2.77 | 9.0E-04 | 2.5E-02 |
| Birc5 | 11799 | 2.76 | 7.4E-05 | 4.9E-03 |
| Hells | 15201 | 2.76 | 2.1E-07 | 6.7E-05 |
| Mfsd2a | 76574 | 2.74 | 5.8E-05 | 4.1E-03 |
| Ckap2l | 70466 | 2.74 | 1.2E-05 | 1.3E-03 |
| Scd1 | 20249 | 2.72 | 1.8E-07 | 6.0E-05 |
| Dtl | 76843 | 2.70 | 4.6E-09 | 4.3E-06 |
| AU018091 | 245128 | 2.68 | 3.1E-09 | 3.5E-06 |
| Plb1 | 665270 | 2.64 | 3.0E-12 | 1.4E-08 |
| Uhrf1 | 18140 | 2.63 | 7.3E-08 | 3.5E-05 |
| Ccnb2 | 12442 | 2.62 | 4.6E-05 | 3.4E-03 |
| Afp | 11576 | 2.61 | 1.8E-03 | 3.9E-02 |
| Cdc20 | 107995 | 2.61 | 2.0E-04 | 9.5E-03 |
| Clec4n | 56620 | 2.58 | 3.6E-05 | 2.9E-03 |
| Tk1 | 21877 | 2.57 | 1.9E-07 | 6.3E-05 |
| Krtap17-1 | 77914 | 2.57 | 4.9E-05 | 3.6E-03 |
| Ccna2 | 12428 | 2.56 | 2.3E-06 | 4.0E-04 |
| Lrg1 | 76905 | 2.53 | 2.2E-06 | 4.0E-04 |
| Anln | 68743 | 2.48 | 3.1E-07 | 9.3E-05 |
| Spc25 | 66442 | 2.47 | 2.2E-05 | 2.0E-03 |
| Arhgap11a | 228482 | 2.46 | 1.4E-05 | 1.4E-03 |
| 2200002D01Rik | 72275 | 2.44 | 5.2E-14 | 6.3E-10 |
| Sprr2a2 | 20755 | 2.43 | 7.9E-07 | 2.0E-04 |
| Fdps | 110196 | 2.42 | 9.4E-09 | 6.9E-06 |
| Psat1 | 107272 | 2.42 | 7.4E-12 | 2.8E-08 |
| Chia | 81600 | 2.40 | 2.9E-07 | 8.9E-05 |
| Spp1 | 20750 | 2.40 | 2.3E-03 | 4.6E-02 |
| Hmmr | 15366 | 2.37 | 2.4E-03 | 4.7E-02 |
| D10Bwg1379e | 215821 | 2.34 | 3.6E-05 | 2.9E-03 |
| Cdc20 | 107995 | 2.34 | 2.0E-04 | 8.2E-03 |
| Tpx2 | 72119 | 2.33 | 1.0E-04 | 7.5E-03 |
| Sgol2 | 68549 | 2.32 | 3.1E-06 | 5.0E-04 |
| Clec4n | 56620 | 2.32 | 4.0E-04 | 1.4E-02 |
| Hells | 15201 | 2.32 | 3.6E-07 | 1.0E-04 |
| Tnc | 21923 | 2.31 | 1.3E-05 | 1.4E-03 |
| Nrcam | 319504 | 2.31 | 4.2E-07 | 1.0E-04 |
| D17H6S56E-5 | 110956 | 2.30 | 2.1E-06 | 4.0E-04 |
| Mal | 17153 | 2.28 | 1.8E-08 | 1.1E-05 |
| Krt15 | 16665 | 2.26 | 2.3E-03 | 4.6E-02 |
| Ncapg | 54392 | 2.25 | 3.8E-05 | 3.0E-03 |
| Lass3 | 545975 | 2.23 | 7.0E-09 | 5.4E-06 |
| Slc26a4 | 23985 | 2.22 | 1.6E-03 | 3.6E-02 |
| Ccna2 | 12428 | 2.21 | 4.3E-07 | 1.0E-04 |
| Clspn | 269582 | 2.22 | 2.7E-08 | 1.5E-05 |
| Serpina3n | 20716 | 2.21 | 1.1E-03 | 3.0E-02 |
| Lamc2 | 16782 | 2.21 | 9.6E-07 | 2.0E-04 |
| Areg | 11839 | 2.20 | 4.2E-05 | 3.2E-03 |
| Plk4 | 20873 | 2.19 | 4.0E-04 | 1.6E-02 |
| Lgals3 | 16854 | 2.19 | 9.7E-13 | 6.3E-09 |
| Gins1 | 69270 | 2.18 | 2.8E-09 | 3.5E-06 |
| Timp1 | 21857 | 2.17 | 9.5E-05 | 5.8E-03 |
| Hist1h2ad | 319171 | 2.18 | 5.6E-05 | 4.0E-03 |
| Retnla | 57262 | 2.15 | 1.3E-08 | 8.8E-06 |
| Aurka | 20878 | 2.15 | 2.0E-04 | 1.0E-02 |
| Mal | 17153 | 2.15 | 8.9E-08 | 3.9E-05 |
| Col3a1 | 12825 | 2.14 | 8.8E-06 | 1.0E-03 |
| Foxn4 | 116810 | 2.14 | 7.0E-04 | 2.2E-02 |
| Pla1a | 85031 | 2.14 | 2.4E-07 | 7.6E-05 |
| Atp6v0d2 | 242341 | 2.12 | 7.0E-04 | 2.1E-02 |
| Cdca8 | 52276 | 2.12 | 2.0E-04 | 9.2E-03 |
| Fam111a | 107373 | 2.11 | 9.0E-04 | 2.5E-02 |
| Lamb3 | 16780 | 2.10 | 7.2E-05 | 4.8E-03 |
| Racgap1 | 26934 | 2.10 | 2.0E-04 | 8.8E-03 |
| Kntc1 | 208628 | 2.09 | 4.3E-06 | 6.0E-04 |
| Mcm5 | 17218 | 2.08 | 2.9E-07 | 8.9E-05 |
| Cdca5 | 67849 | 2.08 | 6.0E-04 | 2.0E-02 |
| Mki67 | 17345 | 2.08 | 5.0E-04 | 1.8E-02 |
| Melk | 17279 | 2.08 | 3.8E-05 | 3.0E-03 |
| Glrx | 93692 | 2.07 | 6.5E-07 | 2.0E-04 |
| Sgol1 | 72415 | 2.07 | 2.8E-06 | 5.0E-04 |
| Cenpk | 60411 | 2.07 | 7.4E-06 | 9.0E-04 |
| Cda | 72269 | 2.06 | 4.4E-09 | 4.3E-06 |
| Dclk1 | 13175 | 2.05 | 2.3E-05 | 2.1E-03 |
| Brca1 | 12189 | 2.04 | 1.7E-06 | 3.0E-04 |
| Cdca3 | 14793 | 2.04 | 8.0E-04 | 2.4E-02 |
| Mpzl3 | 319742 | 2.03 | 9.9E-09 | 7.2E-06 |
| Elovl1 | 54325 | 2.03 | 1.0E-04 | 6.4E-03 |
| Rrm2 | 20135 | 2.02 | 6.3E-06 | 8.0E-04 |
| Fgl1 | 234199 | 2.01 | 1.2E-03 | 3.0E-02 |
| Itgb6 | 16420 | 2.01 | 1.8E-05 | 1.7E-03 |
| Trim59 | 66949 | 2.01 | 3.3E-07 | 9.7E-05 |
| Dclk1 | 13175 | 2.00 | 4.0E-10 | 8.1E-07 |
| Elovl7 | 74559 | 2.04 | 1.1E-05 | 1.2E-03 |
| Bub1 | 12235 | 2.00 | 1.2E-03 | 3.0E-02 |
| Ly6g6c | 68468 | 2.00 | 3.5E-05 | 2.8E-03 |
