# Supplementary material for: Oxidative stress–induced mitochondrial dysfunction drives inflammation and airway smooth muscle remodeling in patients with chronic obstructive pulmonary disease
Source: J Allergy Clin Immunol. 2015 Sep;136(3):769–80. doi: 10.1016/j.jaci.2015.01.046 (PMC4559140; doi:10.1016/j.jaci.2015.01.046)
Supplement: Table E2 [file mmc3.docx]

**Table E2. Differentially expressed genes in ozone-treated lungs; downregulated genes.**

| **Gene Symbol** | **Entrez Gene** | **Ozone vs Air** | **Ozone vs Air** | **Ozone vs Air** |
| --- | --- | --- | --- | --- |
|  |  | **Fold Change** | **P Value** | **FDR** |
| Asgr1 | 11889 | -5.99 | 5.2E-10 | 9.0E-07 |
| AU018778 | 234564 | -4.90 | 4.9E-06 | 7.0E-04 |
| Hmgcs2 | 15360 | -3.73 | 3.1E-11 | 9.8E-08 |
| Bbox1 | 170442.00 | -3.55 | 8.1E-07 | 2.0E-04 |
| Abp1 | 76507 | -3.52 | 9.3E-06 | 1.1E-03 |
| Hmgcs2 | 15360 | -3.47 | 6.5E-10 | 1.1E-06 |
| Slc35d3 | 76157 | -3.43 | 1.2E-08 | 8.4E-06 |
| Adamts20 | 223838 | -3.11 | 9.7E-05 | 5.9E-03 |
| Cd163 | 93671.00 | -2.94 | 7.4E-06 | 9.0E-04 |
| 1700012B09Rik | 69325 | -2.82 | 7.2E-07 | 2.0E-04 |
| Azgp1 | 12007.00 | -2.71 | 2.8E-06 | 5.0E-04 |
| Hs6st2 | 50786 | -2.71 | 6.0E-06 | 8.0E-04 |
| Agrp | 11604 | -2.63 | 3.3E-06 | 5.0E-04 |
| Pcp4l1 | 66425 | -2.63 | 7.8E-08 | 3.6E-05 |
| St8sia1 | 20449 | -2.62 | 3.6E-09 | 3.7E-06 |
| Gm1574 | 100045399 | -2.45 | 2.0E-04 | 1.1E-02 |
| Dcdc2a | 195208 | -2.44 | 2.0E-04 | 1.0E-02 |
| Enpp3 | 209558 | -2.41 | 6.1E-06 | 8.0E-04 |
| 4922501L14Rik | 209601 | -2.40 | 1.0E-04 | 7.4E-03 |
| Diras2 | 68203 | -2.39 | 6.5E-05 | 4.4E-03 |
| Rsph3a | 66832 | -2.38 | 1.0E-05 | 1.1E-03 |
| Iqca | 74918 | -2.37 | 4.0E-04 | 1.4E-02 |
| Tex9 | 21778 | -2.37 | 4.1E-05 | 3.2E-03 |
| Fmo3 | 14262 | -2.36 | 7.4E-05 | 4.9E-03 |
| Scara5 | 71145 | -2.35 | 1.0E-04 | 7.6E-03 |
| Tstd1 | 226654 | -2.30 | 2.6E-06 | 4.0E-04 |
| Iyd | 70337 | -2.28 | 3.7E-05 | 2.9E-03 |
| Col8a2 | 329941 | -2.72 | 1.5E-06 | 3.0E-04 |
| Spag16 | 66722 | -2.30 | 2.0E-04 | 8.2E-03 |
| Shisa9 | 72555 | -2.22 | 2.0E-04 | 9.1E-03 |
| Wif1 | 24117 | -2.14 | 7.0E-05 | 4.7E-03 |
| Pon1 | 18979 | -2.14 | 1.4E-05 | 1.4E-03 |
| Clec10a | 17312 | -2.21 | 1.7E-07 | 5.7E-05 |
| Endog | 13804 | -2.14 | 7.0E-06 | 9.0E-04 |
| Endog | 13804 | -2.14 | 2.9E-06 | 5.0E-04 |
| D2Wsu81e | 227695 | -2.13 | 6.2E-06 | 8.0E-04 |
| Cyp4f15 | 106648 | -2.13 | 2.1E-03 | 4.4E-02 |
| Kcnip4 | 80334 | -2.11 | 3.3E-06 | 5.0E-04 |
| Dnahc6 | 330355 | -2.10 | 6.0E-04 | 2.0E-02 |
| Penk | 18619 | -2.09 | 6.7E-06 | 9.0E-04 |
| Cidea | 12683 | -2.09 | 1.0E-04 | 6.1E-03 |
| Galntl2 | 78754 | -2.08 | 1.5E-07 | 5.3E-05 |
| Fank1 | 66930 | -2.08 | 5.0E-04 | 1.8E-02 |
| Ccl8 | 20307 | -2.07 | 2.5E-03 | 4.9E-02 |
| Ky | 16716 | -2.06 | 2.0E-04 | 8.8E-03 |
| Ttc9 | 69480 | -2.06 | 6.0E-04 | 2.0E-02 |
| Ephx4 | 100046112 | -2.06 | 1.0E-04 | 6.7E-03 |
| Calml4 | 75600 | -2.06 | 1.8E-05 | 1.7E-03 |
| 2010001K21Rik | 69829 | -2.05 | 5.0E-05 | 3.6E-03 |
| Kcnmb2 | 72413 | -2.05 | 6.0E-04 | 2.0E-02 |
| Itih5 | 209378 | -2.05 | 1.5E-07 | 5.3E-05 |
| Nr4a3 | 18124 | -2.04 | 2.6E-03 | 4.9E-02 |
| AI317395 | 215929 | -2.04 | 6.0E-04 | 2.0E-02 |
| Camta1 | 100072 | -2.03 | 1.4E-07 | 5.3E-05 |
| Kcnmb4 | 100047870 | -2.02 | 5.2E-05 | 3.7E-03 |
| Grm4 | 268934 | -2.01 | 5.3E-08 | 2.6E-05 |
| Ptprz1 | 19283 | -2.01 | 5.0E-04 | 1.7E-02 |
| Chek2 | 50883 | -2.01 | 1.1E-05 | 1.2E-03 |
| Ass1 | 11898.00 | -2.00 | 5.8E-09 | 4.7E-06 |
| Kcnmb2 | 72413 | -2.05 | 6.0E-04 | 2.0E-02 |
| Itih5 | 209378 | -2.05 | 1.5E-07 | 5.3E-05 |
| Nr4a3 | 18124 | -2.04 | 2.6E-03 | 4.9E-02 |
| AI317395 | 215929 | -2.04 | 6.0E-04 | 2.0E-02 |
| Camta1 | 100072 | -2.03 | 1.4E-07 | 5.3E-05 |
| Kcnmb4 | 100047870 | -2.02 | 5.2E-05 | 3.7E-03 |
| Grm4 | 268934 | -2.01 | 5.3E-08 | 2.6E-05 |
| Ptprz1 | 19283 | -2.01 | 5.0E-04 | 1.7E-02 |
| Chek2 | 50883 | -2.01 | 1.1E-05 | 1.2E-03 |
| Ass1 | 11898.00 | -2.00 | 5.8E-09 | 4.7E-06 |
