# Supplementary figures and images for: Oxidative stress–induced mitochondrial dysfunction drives inflammation and airway smooth muscle remodeling in patients with chronic obstructive pulmonary disease
Source: J Allergy Clin Immunol. 2015 Sep;136(3):769–80. doi: 10.1016/j.jaci.2015.01.046 (PMC4559140; doi:10.1016/j.jaci.2015.01.046)

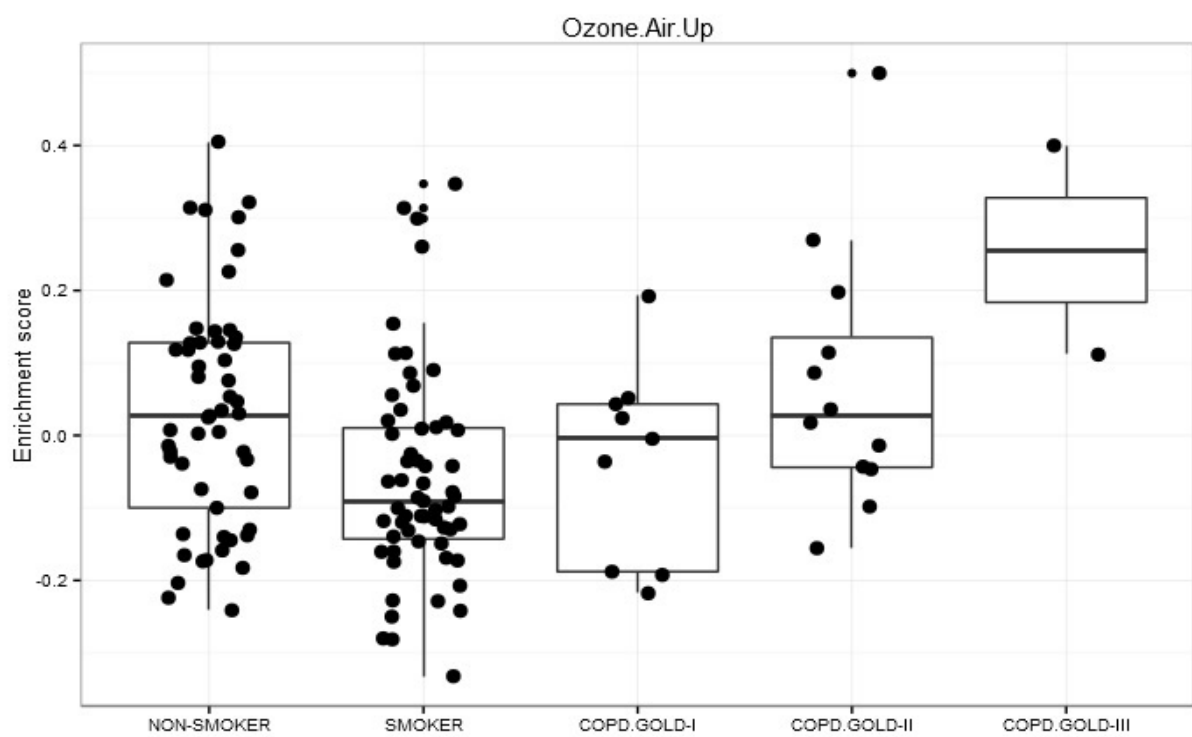

Supplement: Fig E1 [file mmc4.pdf]

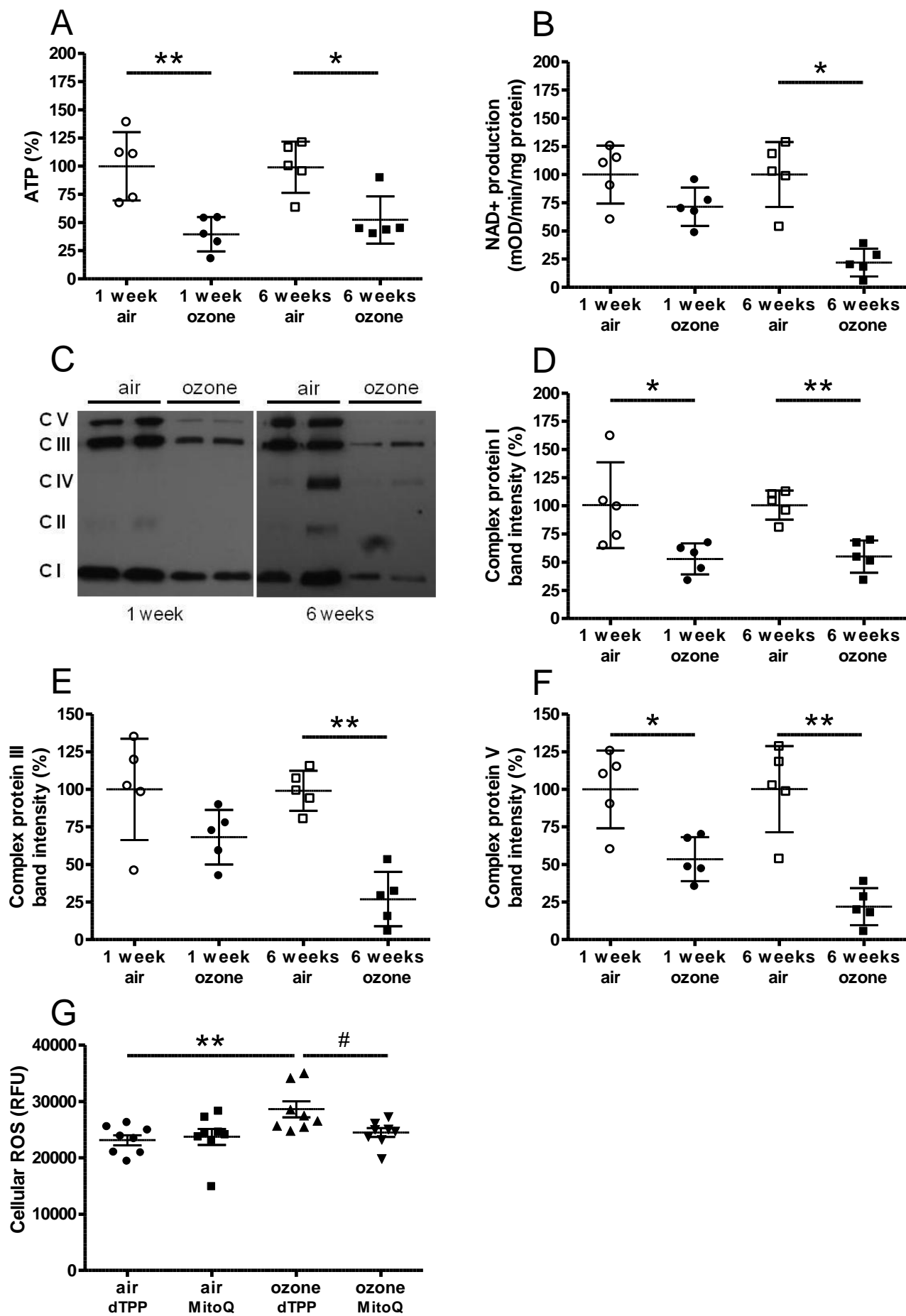

Supplement: Fig E2 [file mmc5.pdf]

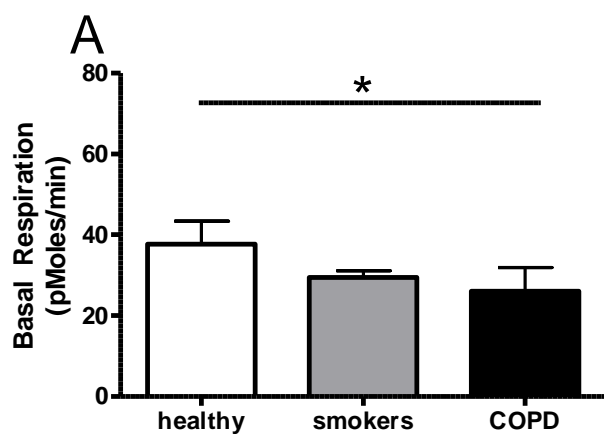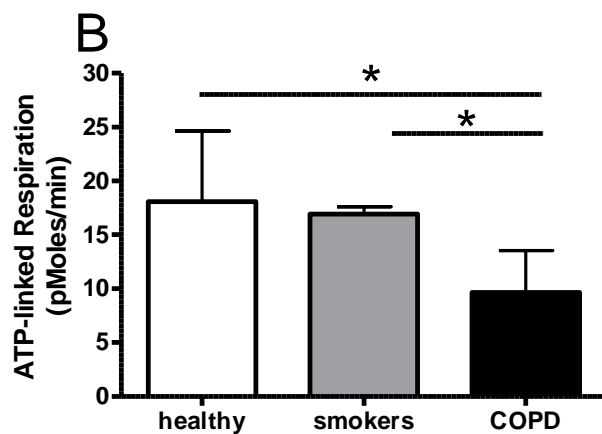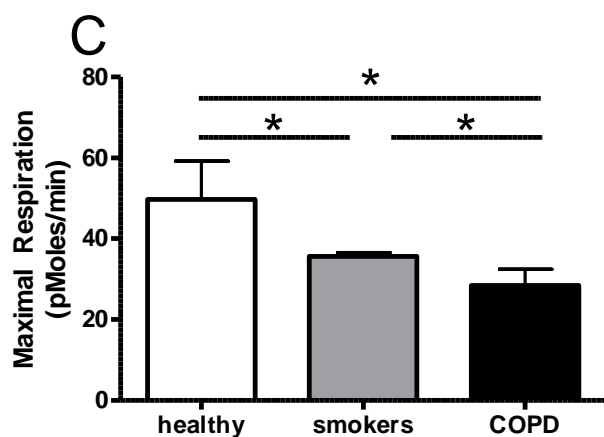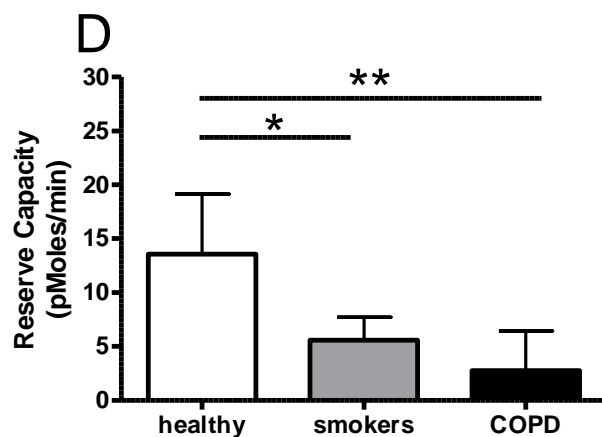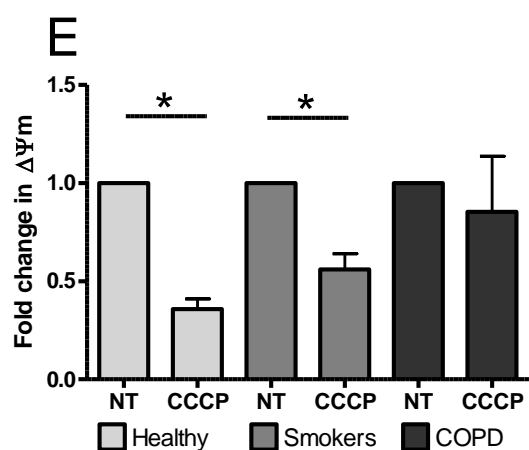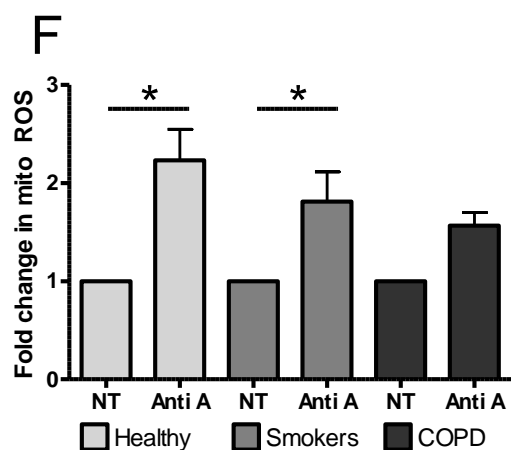

Supplement: Fig E3 [file mmc6.pdf]

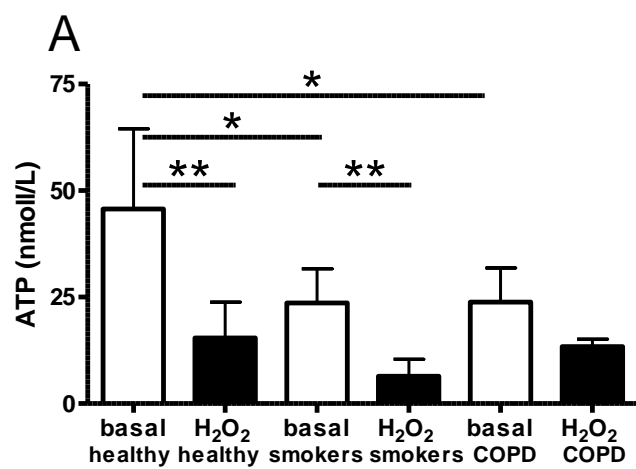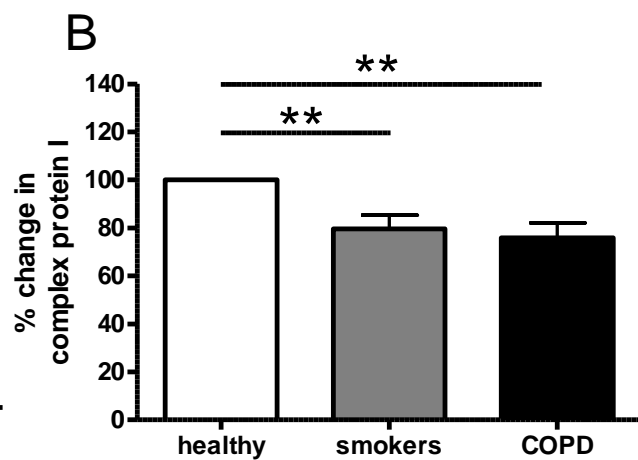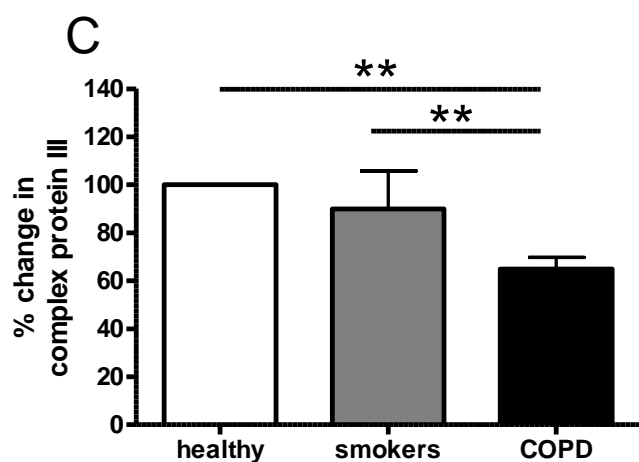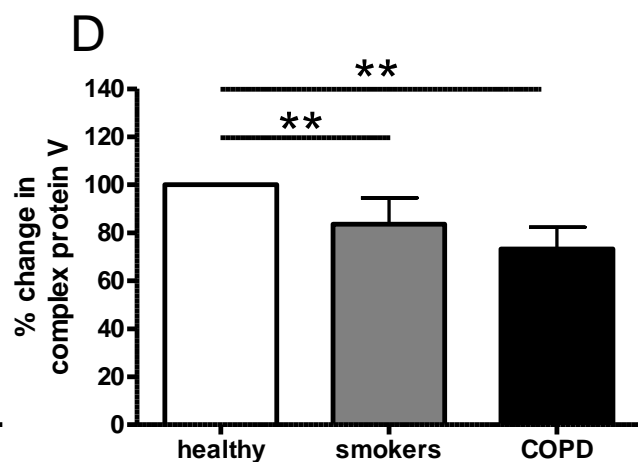

Supplement: Fig E4 [file mmc7.pdf]
